# ZNF655 accelerates progression of pancreatic cancer by promoting the binding of E2F1 and CDK1

**DOI:** 10.1038/s41389-022-00418-2

**Published:** 2022-08-04

**Authors:** Zhuo Shao, Chenggang Li, Qiao Wu, Xingmao Zhang, Yang Dai, Shenming Li, Xinyuan Liu, Xinying Zheng, Jiansheng Zhang, Hua Fan

**Affiliations:** 1 Department of Hepatobiliary and Pancreatic Surgery, Changhai Hospital affiliated to Naval Medical University, Shanghai, China; 2 State Key Laboratory of Medicinal Chemical Biology & College of Pharmacy, Nankai University, Tianjin, China; 3 Department of Hepatobiliary and Pancreatospleenic Surgery, Beijing Chaoyang Hospital, Capital Medical University, Beijing, 100020 China; 4 Department of Hepatobiliary and Pancreatic Surgery, The Second Hospital of Hebei Medical University, Shijiazhuang, China

**Keywords:** Pancreatic cancer, Molecular biology

## Abstract

Pancreatic cancer has an extremely terrible prognosis and is a common cause of cancer death. In this study, the clinic value, biological function and underlying mechanisms of Zinc finger protein 655 (ZNF655) in human pancreatic cancer were evaluated. The expression level of ZNF655 in pancreatic cancer was determined by immunohistochemistry (IHC) staining. The biological effects of ZNF655 in pancreatic cancer cells was investigated by loss/gain-of-function assays in vitro and in vivo. The downstream molecular mechanism of ZNF655 was explored using co-immunoprecipitation (Co-IP), dual-luciferase reporter and chromatin immunoprecipitation (Ch-IP). ZNF655 expression was significantly elevated in human pancreatic cancer and possessed clinical value in predicting poor prognosis. Functionally, ZNF655 knockdown inhibited the biological progression of pancreatic cancer cells, which was characterized by weaken proliferation, enhanced apoptosis, arrested cell cycle in G2, impeded migration, and suppressed tumor growth. Mechanistically, ZNF655 played an important role in promoting the binding of E2F transcription factor 1 (E2F1) to the cyclin-dependent kinase 1 (CDK1) promoter. Furthermore, knockdown of CDK1 alleviated the promoting effects of ZNF655 overexpression in pancreatic cancer cells. The promotive role of ZNF655 in pancreatic cancer via CDK1 was determined, which drew further interest regarding its clinical application as a promising therapeutic target.

## Introduction

Pancreatic cancer is a malignancy of the digestive tract that originating from the glandular epithelium, responsible for substantial morbidity and mortality worldwide [1]. The sobering reality is that pancreatic cancer has an extremely poor prognosis, with a 5-year survival rate of only about 10%, and it is becoming a common cause of cancer death [2]. Pancreatic cancer is generally classified into four types according to the degree of disease: resectable, marginally resectable, locally advanced and metastatic [3]. For a small number of patients diagnosed with resectable tumors, surgical resection is a great opportunity of curing the disease [4]. Unfortunately, only 20% of patients can survive 5 years after surgery [5]. Poor prognosis and widespread drug resistance continue to plague pancreatic cancer patients. Recently, with the development of genomics and proteomics, the exploration of targeted drugs for precision medicine has become a hot spot. For instance, targeted medicine that overexpress or mutation-related pathway dysfunction of epidermal growth factor receptor (EGFR) [6], vascular endothelial growth factor (VEGF) [7, 8], Notch [9], Hedgehog [10], mitogen-activated protein extracellular signal-regulated kinase (MEK) [11], and KRAS [12] common in pancreatic tumors are being extensively investigated. Although trials of these novel combination therapies have been modestly successful in the clinic, the overall survival of pancreatic cancer has been little improved [13]. To that end, further exploration on pancreatic cancer is extremely important for the identification of novel targets.

Zinc finger (ZNF) proteins are the largest family of transcription factors in the human genome and have a variety of molecular functions [14]. The different combinations of ZNF motifs lead to diverse functions of ZNF proteins in biological processes such as development, differentiation, metabolism and autophagy [15, 16]. Over the past few decades, accumulating evidence has been shed on the potential role of ZNF proteins in cancer progression [17]. However, the underlying mechanism of zinc finger protein in cancer varies among different cancer types [18]. For the most part, Cys2His2 ZNF proteins act as trans regulator of gene expression, which exert key role in cellular processes such as cell development, differentiation and inhibition of malignant cell transformation [19]. Numerous reports have shown a direct relationship between apoptosis resistance, chemotherapy resistance and increased expression of ZNF703. Moreover, ZNF703 has oncogene effects in breast cancer [20]. In addition, reduced expression of ZNF233 in hepatocellular carcinoma inhibits cell proliferation and tumorigenesis [21]. In contrast, ZNF554 is regarded as a potential tumor suppressor in malignant gliomas, and its reduced expression may lead to the loss of oncogene suppression and the activation of tumor pathways [22]. As a transcription factor of Cys2His2 type ZNF, the role of ZNF655 in pancreatic cancer has not been reported.

At present study, we evaluated the expression level, biological function and underlying mechanisms of ZNF655 in human pancreatic cancer. ZNF655 was abundantly expressed in pancreatic cancer, and its high expression was significantly correlated with poor prognosis. Reduced expression of ZNF655 resulted in reduced malignant phenotypes in pancreatic cancer cells. Mechanistically, ZNF655 played an important role in promoting the binding of E2F1 to the CDK1 promoter. Furthermore, knockdown of CDK1 alleviated the promoting effects of ZNF655 overexpression in pancreatic cancer cells. These findings suggested that ZNF655 had a stimulative effect on pancreatic cancer via CDK1 and was a candidate target for molecular therapy.

## Materials and methods

### Pancreatic cancer tissue collection and cell culture

Human survival pancreatic cancer tissues (*n* = 97) and paired pancreatic tissues (*n* = 69) were collected during surgical resection at Beijing Chaoyang hospital. The experimental procedures of this study were approved by the ethics committee of Beijing Chaoyang hospital (No. 2019-S-243) and all patients signed an informed consent form. Pancreatic cancer cell lines (PANC-1, SW1990 and BXPC-3) and normal pancreatic cell line HPDE6-C7 were purchased from Cell Bank of the Chinese Academy of Sciences (Shanghai, China). The cells were cultured in DMEM supplemented with 10% fetal bovine serum (Invitrogen Gibco) and maintained in a 37 °C, 5% CO_2_ incubator.

### Immunohistochemistry (IHC) staining

Formalin-immobilized tissues were dewaxed in xylene, rehydrated in ethanol solution, incubated with 3% hydrogen peroxide to block endogenous peroxidase and nonspecific binding sites. The tissues were incubated with the primary anti-ZNF655 (1:200, Invitrogen, PA5-56183), anti-CDK1 (1:50, Sigma, HPA003387) antibody at 4 °C overnight and then with the secondary antibody HRP goat anti-rabbit IgG (1:200, Beyotime, A0208) at room temperature for 1 h. After incubation with peroxidase conjugated streptavidin and diaminobenzidine, hematoxylin was stained. The staining intensity was scored according to the criteria described in the literature [23]. The result greater than or equal to median values of IHC was defined as ZNF655 high expression, otherwise low expression.

### Lentivirus transfection and cell infection

Three shRNAs against ZNF655 or CDK1 interference sequences were synthesized (shZNF655-1, 5'-GCCCAGGAAGCAGCAGGGTCA-3'; shZNF655-2, 5'-CACCGACATGGAACAGGGACT-3'; shZNF655-3, 5'-TCCAGTTTCAGTCTTTGGAGA-3'; shCDK1-1, 5'-TTCCATGGATCTGAAGAAATA-3'; shCDK1-2, 5'-AGACTAGAAAGTGAAGAGGAA-3'; shCDK1-3, 5'-ATGGAGTTGTGTATAAGGGTA-3') and the lowest expression interference sequence of ZNF655 or CDK1 was selected, respectively. The fragment was inserted into lentiviral vector BR-V108 with green fluorescent protein (GFP) (BIOSCIRES). The 293 T cells were co-transfected with 10 μg recombinant BR-V108 vector and virus packaging plasmid (7.5 μg pMD2.G and 5 μg pSPAX2) (BIOSCIRES) for 72 h. Meanwhile, the amplified sequence of ZNF655 (ZNF655) was synthesized and inserted into lentiviral vector LV-003 (BIOSCIRES) to construct overexpression of ZNF655. After that, 2 × 10^5^ PANC-1 and SW1990 cells were cultured for 24 h and infected with lentivirus shZNF655, shCDK1 and ZNF655 (1 × 10^8^ TU/mL) at a MOI (multiplicity of infection) of 10. Finally, the expression of GFP was observed under fluorescence microscope (OLYMPUS) 72 h after lentivirus infection.

### RNA extraction and qPCR

PANC-1 and SW1990 cells RNA was purified with Trizol [24] and reverse transcribed into cDNA using the Maxima First Strand cDNA Synthesis Kit (Thermo Fisher Scientific). Quantification of mRNA expression levels of ZNF655 and CDK1 was accomplished by SYBR Green master mix (Thermo Fisher Scientific) by ABI Prism 7500 sequence detection system (Applied Biosystems) with normalization to the expression of GAPDH. The primer sequences were listed in Table S1.

### Western blotting (WB) and Co-immunoprecipitation (Co-IP) assay

PANC-1 and SW1990 cells protein was purified with RIPA (Beyotime) and the concentration was detected using BCA Protein Assay Kit (Beyotime). The 20 μg/well total protein was subjected to 10% SDS–PAGE, transferred to PVDF membrane (Millipore), hybridized with corresponding primary antibody (Table S2) overnight at 4 °C, incubated with secondary antibody at room temperature for 2 h. After that, protein signal was visualized through chemiluminescence ECL kit (Thermo Fisher Scientific) and GAPDH as load control. ZNF655 and CDK1 protein–protein interaction was analyzed by Co-IP assay and the experimental procedures were performed as previously described [25].

### MTT and cell counting assay

PANC-1 and SW1990 cells were cultured in 96-well plates at a density of 2000 cells/well. Cell viability was detected by MTT assay for 5 days [26] and cell proliferation ability was analyzed by drawing growth curve. The cells with GFP were identified with Celigo, photographed, counted, and the cell growth curve was plotted for 5 days.

### Detection of cell cycle and apoptosis

PANC-1 and SW1990 cells were inoculated into 6-well plates (2 mL/well) for 5 days.

For apoptosis analysis, the cell precipitates were successively washed by precooled D-hanks (pH = 7.2~7.4) and 1×binding buffer (eBioscience), resuspended by 200 μL 1× binding buffer, stained with 10 μL Annexin V-APC (eBioscience) at room temperature in the dark for 15 min and detected by flow cytometry (Millipore). For cell cycle analysis, the cells were centrifuged for 5 min, the cell precipitates were eluted with precooled PBS (pH = 7.2~7.4), fixed with 70% ethanol for at least 1 h, stained with PI (Sigma) and monitored by flow cytometry.

### Human apoptosis antibody array assay

PANC-1 cells protein was purified with RIPA (Beyotime) and the concentration was detected using BCA Protein Assay Kit (Beyotime). The concentrations of 43 kinds of human apoptotic markers in cell lysate were simultaneously detected in strict accordance with the instructions of the kit (Human Apoptosis Antibody Array–Membrane, Abcam). After that, the signal of apoptotic markers was visualized through chemiluminescence ECL kit (Thermo Fisher Scientific) and quantified by Image J software (National Institutes of Health).

### Wound-healing assay

PANC-1 and SW1990 cells were cultured in 6-well plates (100 μL/well) at a density of 4000 cells/well. The specific experimental procedures followed the description in the literature [27]. In order to avoid the influence of cell proliferation on migration, the cells were placed in a serum-free medium and treated with mitomycin for 1 h before performing wound-healing experiments. A line wound was drawn by a pipette tip across the cell layer. The cells were washed with PBS, fixed with 3.7% paraformaldehyde (Corning) for 15 min, stained with 1% crystal violet (Corning) for 10 min. The cells were viewed under a microscope for image acquisition and Image J software (National Institutes of Health) was used to quantify the distance (μm) between the scratches at 0 h, 8 h and 72 h.

### Transwell assay

PANC-1 and SW1990 cells at a density of 80,000 cells/well were cultured into Transwell chambers (24-well, 8-mm pore, Corning) for 24 h at 37 °C, of which 100 μL cell suspension in the inner compartment and 500 μL DMEM medium containing 30% FBS in the outer compartment. The non-invading cells on the upper chamber were removed, while the cells adhering to the Polycarbonate membrane were fixed with 4% precooled paraformaldehyde for 30 min and stained with 0.1% crystal violet for 20 min at room temperature. Finally, the cells were photographed from five randomly selected fields under a 200× microscope.

### Gene microarray

After PANC-1 cells infected with lentivirus shZNF655 and shCtrl, RNA was purified and sequenced through Affymetrix human Gene Microarray Prime View (Affymetrix Scanner 3000 scan) to recognize differentially expressed genes (DEGs). DEGs was selected with criterion of |Fold Change | ≥ 1.3 and false discovery rate (FDR) ≤ 0.05 and presented as a volcano plot and hierarchical clustering. Significant enrichment of DEGs in classical pathways, disease and function, and interaction networks was explored based on Ingenuity Pathway Analysis (IPA).

### Dual-luciferase reporter assay

The CDK1 promoter region was amplified and the fragment was cloned into the luciferase reporter vector GL002 (Promega Madison, USA), named as GL002- CDK1. Mutant construct GL002-CDK1-Mut was generated by site-directed mutagenesis. Luciferase assay was performed as described previously [28]. Each experimental analysis was repeated three times.

### Chromatin immunoprecipitation (Ch-IP) assay

According to the manufacturer’s instructions, Ch-IP assay was performed using the Simple ChIP^®^ Enzymatic Chromatin IP Kit (Cat No, 9002 S, CST, USA). In brief, the cells were transfected with ZNF655-overexpressing vector or its empty vector and incubated for 48 h at 37 °C with 5% CO_2_. Subsequently, the cells were crosslinked with 37% formaldehyde followed by lysed in SDS buffer and sheared sonication to fragment the DNA. Afterwards, the sonicated chromatin was precipitated by incubating it with according antibody overnight at 4 °C. The Protein–DNA complexes were then purified and the purified DNA was dissolved in nuclease-free water followed by qPCR analysis using the primers of CDK1 promoter and SYBR Green I Master (Roche, USA).

### In vivo xenograft model of mice

The experimental procedures performed on mice were approved by the Ethics Committee of Beijing Chaoyang Hospital and the Animal Protection Association. The total of 20 BALB/c nude SPF mice (18–22 g, 4–6 weeks) were obtained from Viton Lihua Laboratory Animal Technology Co., Ltd. The 500 μL SW1990 cells infected with lentivirus shZNF655 (*n* = 5) and shCtrl (*n* = 5) were subcutaneously injected into the right armpit of mice (1 × 10^7^ cells/mouse), respectively. Tumor volumes of mice was monitored 7 days after injection and then collected once or twice a week. Notably, the tumor volume was calculated using the formula π/6 × *L* × *W* × *W* (*L*: longest dimension, *W*: dimension perpendicular to length). After 26 days, mice were sacrificed by intraperitoneal injection of 0.7% pentobarbital sodium at a dose of 10 μL/g, and the tumors were removed for taking photos and weighting. Finally, the signal intensity of ZNF655 and CDK1 in mice tumor tissues was determined by IHC staining.

### Statistical analysis

All data were presented as means ± SD and *P* < 0.05 were considered statistically significant. Comparisons between different groups were conducted by Student’s *t*-test or ANOVA as appropriate. The clinical relevance between ZNF655 and clinicopathological characteristics of pancreatic cancer patients was analyzed by univariate and multivariate analyses and Kaplan–Meier method.

## Results

### ZNF655 is highly expressed in human pancreatic cancer and predicts poor prognosis

**Fig. 1:**
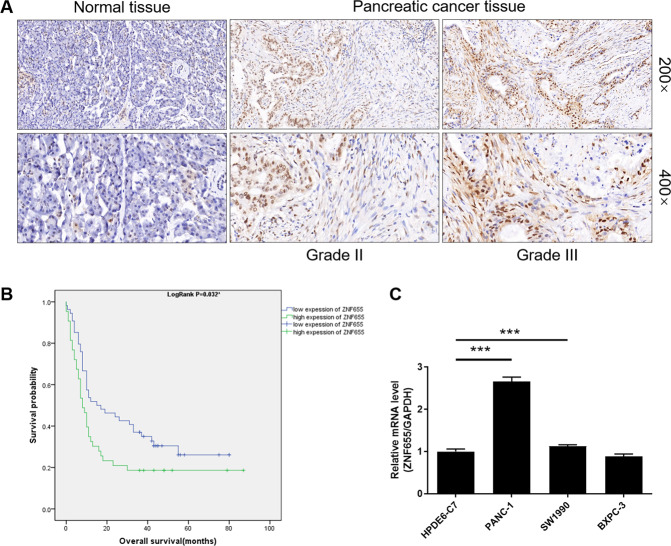
ZNF655 expression is significantly elevated in human pancreatic cancer and possesses clinical value in predicting poor prognosis. **A** The expression level of ZNF655 in pancreatic cancer was determined by IHC staining and representative images were shown. Magnification is 200 and 400. **B** The clinical relevance between ZNF655 and Overall survival of pancreatic cancer patients was analyzed by Kaplan–Meier method. *P = 0.032. **C** The mRNA expression level of ZNF655 in pancreatic cancer cell lines (PANC-1, SW1990 and BXPC-3) and normal pancreatic cell line HPDE6-C7 was identified.

To clarified the clinical relevance of ZNF655 in the progression of human pancreatic cancer, we first examined expression level of ZNF655 in primary pancreatic cancer samples by IHC staining. The signal expression intensity of ZNF655 in pancreatic cancer tissues was significantly higher than that in adjacent normal pancreatic tissues (Fig. 1A, Table 1). According to the eighth edition of the American Joint Committee on Cancer (AJCC) and the Union for International Cancer Control (UICC), the clinical relevance between ZNF655 and clinicopathological characteristics of pancreatic cancer patients was analyzed. High expression of ZNF655 was observed in 21 of 66 low-grade pancreatic cancer (WHO II; 13.13%) and in 22 of 31 high-grade pancreatic cancer (WHO III–IV; 70.97%) (Table 2), suggesting that increased ZNF655 expression was found in higher tumor grade. Furthermore, correlation analysis between the expression level of ZNF655 (high and low) and the overall survival of patients with pancreatic cancer (Grade II, III and IV) was performed using Kaplan–Meier survival. As the expression of ZNF655 increased, the survival time was shortened, suggesting that higher expression of ZNF655 predicted a worse prognosis in patients with pancreatic cancer (Fig. 1B). Collectively, ZNF655 expression was significantly elevated in human pancreatic cancer and had clinical value in predicting poor prognosis.

**Table 1:** Expression patterns in pancreatic cancer tissues and para-carcinoma tissues revealed in immunohistochemistry analysis.

ZNF655 expression	Tumor tissue		Para-carcinoma tissue		*P*-value
	Cases	Percentage	Cases	Percentage	
Low	54	55.7%	56	81.2%	0.001
High	43	44.3%	13	18.8%

**Table 2:** Relationship between ZNF655 expression and tumor characteristics in patients with pancreatic cancer.

Features	No. of patients	ZNF655 expression		*p*-value
		low	high	
All patients	97	54	43	
Age (years)				0.838
≤59	48	27	21	
>59	48	26	22	
Gender				0.390
Male	61	36	25	
Female	36	18	18	
Lymph node positive				0.371
<1	47	23	24	
≥1	41	24	17	
Tumor size				0.509
≤4 cm	59	31	28	
>4 cm	37	22	15	
Grade				0.001
II	66	45	21	
III	30	9	21	
IV	1	0	1	
Stage				0.808
1	37	20	17	
2	55	31	24	
4	2	0	2	
T Infiltrate				0.599
T1	3	3	0	
T2	73	39	34	
T3	20	11	9	
lymphatic metastasis (N)				0.419
N0	50	25	25	
N1	41	24	17	
History of diabetes				0.071
no	5	5	0	
yes	61	35	25	

### Knockdown of ZNF655 inhibits proliferation, promotes apoptosis and impedes migration of pancreatic cancer cells

**Fig. 2:**
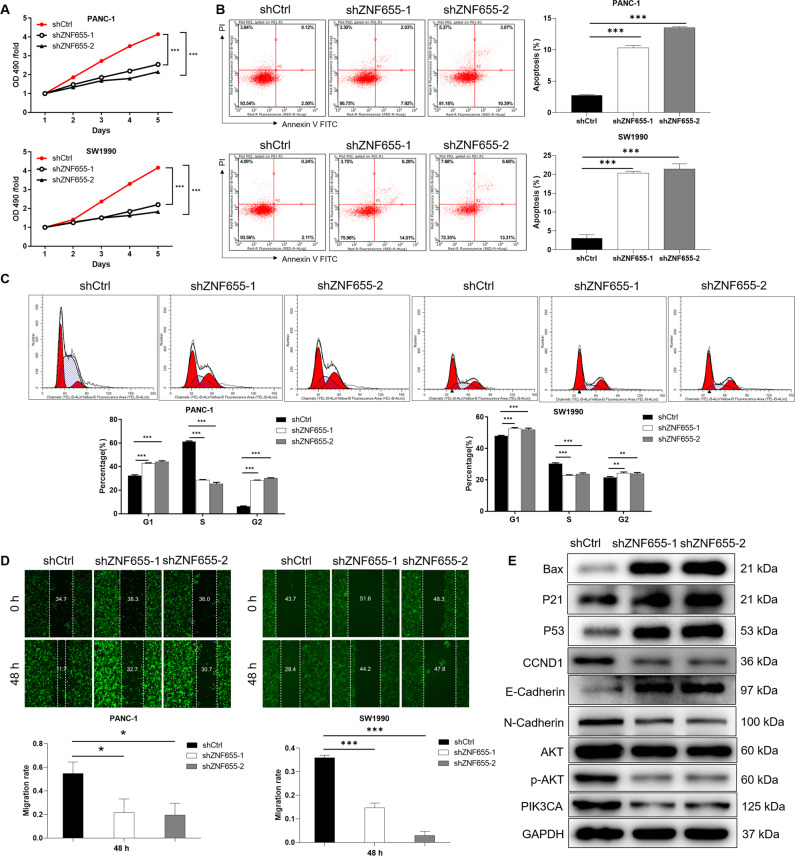
Effects of knockdown of ZNF655 on proliferation, apoptosis, cycle and migration of pancreatic cancer cells. **A** The proliferation of PANC-1 and SW1990 cells after knockdown of ZNF655 was measured using MTT assay. **B**, **C** Cell apoptosis and cycle of PANC-1 and SW1990 cells after knockdown of ZNF655 was analyzed by flow cytometry. **D** The migration of PANC-1 and SW1990 cells after knockdown of ZNF655 was measured using wound-healing assay. **E** The expression of typical proteins related to apoptosis, cycle and migration was detected in pancreatic cancer cells. shCtrl indicates PANC-1 and SW1990 cells infected with a vector-expressing GFP; shZNF655 indicates ZNF655 knockdown in PANC-1 and SW1990 cells. The presented results were representative of experiments repeated at least three times. Data was represented as mean ± SD. *P < 0.05, **P < 0.01, ***P < 0.001.

Additionally, the mRNA expression level of ZNF655 in pancreatic cancer cell lines PANC-1 and SW1990 was significantly higher than that of normal pancreatic cell line HPDE6-C7 (Fig. 1C). In order to further determine the biological functions of ZNF655 in human pancreatic cancer, a series of cell functional experiments were carried out in vitro. The sequences of shRNA targeting ZNF655 (shZNF655-1/2/3) were synthesized and the two sequences with reduced expression of ZNF655 (shZNF655-1/2) were screened out (Fig. S1A). Compared with shCtrl, the protein expression of ZNF655 in PANC-1 and SW1990 cells was significantly reduced after the lentivirus shZNF655-1 and shZNF655-2 were transfected (Fig. S1B). Subsequently, knockdown of ZNF655 in PANC-1 and SW1990 cells was used to investigate the role of ZNF655 in pancreatic cancer. Cell growth curves based on the MTT assay demonstrated that ZNF655 knockdown significantly decreased cell viability and proliferation in PANC-1 and SW1990 cells (Fig. 2A). Furthermore, we examined the cell apoptosis and cycle progression in response to downregulation of ZNF655. The apoptotic index of PANC-1 and SW1990 was significantly increased after ZNF655 expression was reduced, as detected by flow cytometry (Fig. 2B). At the same time, we observed that knockdown of ZNF655 in PANC-1 and SW1990 cells with an accumulation of cells in the G2 phase and a decrease in the S phase (Fig. 2C). In wound-healing assays, the PANC-1 and SW1990 cells transfected with shZNF655-1 and shZNF655-2 lentivirus displayed impairment of migration ability when compared with cells transfected with shCtrl (Fig. 2D). These findings demonstrated that the proliferation and migration were inhibited, cell cycle was arrested in G2, while the apoptosis was enhanced in ZNF655-knockdown PANC-1 and SW1990 cells.

### Knockdown of ZNF655 regulates the expression of typical proteins related to apoptosis, cycle and migration

In addition, the protein expression of human apoptosis signaling pathway related regulator was detected in PANC-1 cells with reduced ZNF655 expression. Compared with the control group, the expression of Bax (BCL2 associated X), BIM (BCL2 like 11), Caspase3, cytoC (cytochrome c), IGFBP-6 (insulin-like growth factor binding protein-6), p21, p27 and p53 was upregulated in shZNF655 group, while the expression of Survivin, IGFBP-2 (insulin-like growth factor binding protein-2) and STNF-R2 (soluble tumor necrosis factor-receptor 2) was downregulated (Fig. S2C). Moreover, the expression of typical proteins related to apoptosis, cycle and migration was detected in pancreatic cancer cells. The results showed that the knockdown of ZNF655 upregulated Bax, p21, p53, and downregulated CCND1, E-cadherin, and N-cadherin (Fig. 2E). Previous study reported that phosphatidylinositol 3-kinase (PI3K)/AKT signaling pathway is one of the most downregulated pathways in human cancers, which is involved in a variety of carcinogenic processes, including cell proliferation, growth, survival, apoptosis and metastasis [29]. In this study, WB results showed that ZNF655 knockdown resulted in downregulation of p-AKT and PIK3CA (Fig. 2E).

### ZNF655 promotes the binding of E2F1 to CDK1 promoter in pancreatic cancer cells

**Fig. 3:**
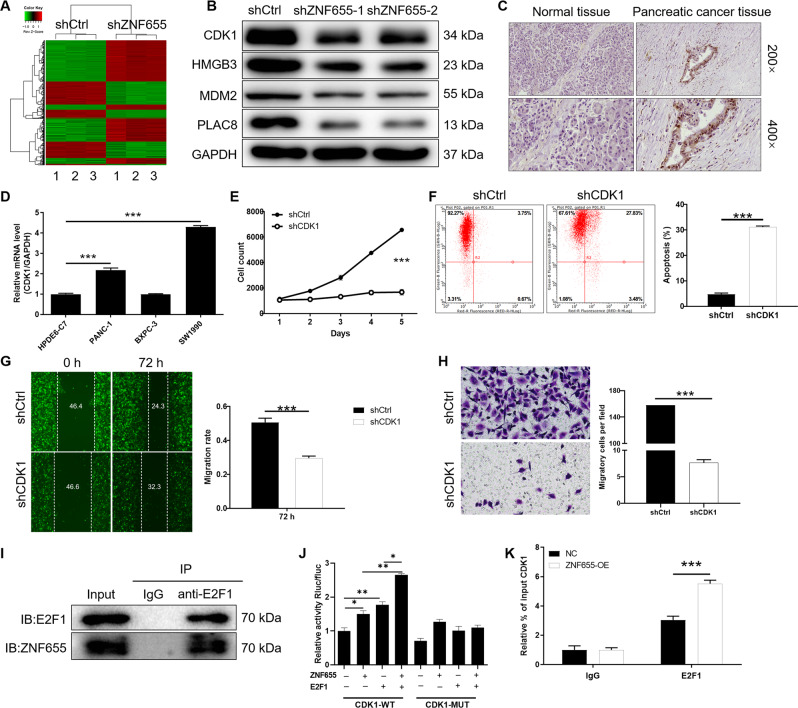
ZNF655 promotes the binding of E2F1 to CDK1 promoter in pancreatic cancer cells. **A** The DEGs between shZNF655 and shCtrl groups of PANC-1 cells were identified. In the heat map of cluster analysis, each column represents a sample and each row represents a differential gene. The red indicates that the gene expression is upregulated, the green indicates that the gene expression is downregulated, the black indicates that the gene expression is not significantly changed, and the gray indicates that the signal strength of the gene is not detected. **B** The expression of several selected DEGs of PANC-1 cells after knockdown of ZNF655 was measured by WB. **C** The expression level of CDK1 in pancreatic cancer was detected by IHC staining and representative images were shown. Magnification is 200 and 400. **D** The mRNA expression level of CDK1 in pancreatic cancer cell lines (PANC-1, SW1990 and BXPC-3) and normal pancreatic cell line HPDE6-C7 was identified by qPCR. **E**–**H** Effects of knockdown of CDK1 on proliferation, apoptosis and migration of pancreatic cancer cells. shCtrl indicates PANC-1 cells infected with a vector-expressing GFP; shCDK1 indicates CDK1 knockdown in PANC-1 cells. **I** Cell lysates from PANC-1 cells were subjected to Co-IP, and anti-E2F1 antibody was used for IP (immunoprecipitation). IgG antibody, a negative control group, were designed to exclude nonspecific immune responses. Input, positive control, refers to using the lysate before IP as IB (immunoblotting) to exclude false positive interference. **J** We mutated the promoter region of CDK1 and performed a dual-luciferase reporter experiment to observe the effects of ZNF655 and E2F1 on the CDK1 promoter activity. **K** Chromatin immunoprecipitation (Ch-IP) assay confirmed that overexpression of ZNF655 induced E2F1 to be recruited to the CDK1 promoter region. The presented results were representative of experiments repeated at least three times. Data was represented as mean ± SD. *P < 0.05, **P < 0.01, ***P < 0.001.

Furthermore, gene microarray results showed that ZNF655 knockdown resulted in 933 upregulated genes and 1430 downregulated genes (Fig. 3A). Additionally, significant enrichment of DEGs in disease and function was explored based on IPA (Fig. S2A), such as Cancer, Organismal Injury and Abnormalities, Cell Death and Survival. Fig. S2B showed the significant enrichment of DEGs in the classical pathway, such as TNFR2 signaling, Cell Cycle: G2/M DNA Damage Checkpoint Regulation. Moreover, the interaction network between ZNF655 and Cell Cycle: G2/M DNA Damage Checkpoint Regulation pathway was illustrated in the Fig. S2C. Accordingly, the expression levels of the first 19 DEGs most significantly enriched in these functions and pathways were further detected by qPCR and WB, respectively (Figs. S2D, 3B). We found that knockdown of ZNF655 downregulated CDK1. Considering that CDK1 is a typical cell cycle regulator and has promoted the progression of pancreatic cancer [30]. Consistently, the expression of CDK1 in pancreatic cancer tissues was higher than that in adjacent normal tissues, suggesting that CDK1 may exhibit an important role in pancreatic cancer. Additionally, the mRNA expression level of CDK1 in pancreatic cancer cell lines PANC-1 and SW1990 was significantly higher than that of normal pancreatic cell line HPDE6-C7 (Fig. 3D). Furthermore, we stably knocked down CDK1 in PANC-1 cells using lentiviral shCDK1 (Fig. S2E) and the loss-of-function results showed a significant inhibition in the proliferation and migration of CDK1-knocked-down PANC-1 cells (Fig. 3E–H).

Combined with our data, we inferred that there may be a connection between ZNF655 and CDK1. Moreover, it is reported that E2F1 binds to the upstream region of CDK1 to accelerate transcription and upregulate its protein expression [31]. Here, cell lysates from PANC-1 cells were subjected to Co-IP, and anti-E2F1 antibody was used for IP. IgG antibody, a negative control group, were designed to exclude nonspecific immune responses. Interestingly, the present study indicated that there was interaction between ZNF655 and E2F1 (Fig. 3I). Therefore, we hypothesized that ZNF655 regulated CDK1 expression through E2F1. Subsequently, we mutated the promoter region of CDK1 and performed a dual-luciferase reporter experiment to observe the effects of ZNF655 and E2F1 on the CDK1 promoter activity. E2F1 could significantly enhance the luciferase activity of CDK1-WT but not CDK1-MUT, suggesting that presence of combination between E2F1 and CDK1. Moreover, overexpression of ZNF655 and E2F1 significantly enhance the activity of CDK1. The results indicated that overexpression of ZNF655 significantly promoted the combination of E2F1 and CDK1 (Fig. 3J). Ch-IP assay further confirmed that overexpression of ZNF655 induced E2F1 to be recruited to the CDK1 promoter region (Fig. 3K). Taken together, these data suggested that ZNF655 facilitated malignant behaviors of pancreatic cancer cells via promoting the binding of E2F1 to CDK1 promoter.

### Knockdown of CDK1 alleviates the promoting effects of ZNF655 overexpression in pancreatic cancer cells

**Fig. 4:**
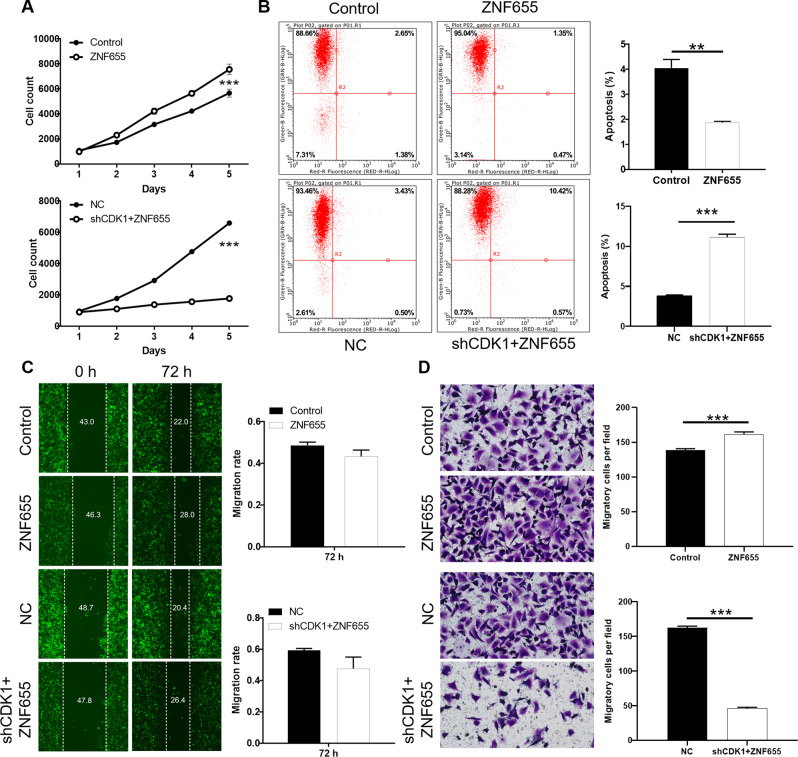
Knockdown of CDK1 alleviates the promoting role of ZNF655 overexpression in pancreatic cancer cells. Detection of alteration in proliferation (**A**), migration (**B**, **C**), apoptosis (**D**) after lentivirus ZNF655 and shCDK1 + ZNF655 infects PANC-1 cells. Control indicates PANC-1 cells infected with empty vector LV-003, as negative control; ZNF655 indicates ZNF655 overexpression in PANC-1 cells; NC(KD + OE) indicates PANC-1 cells infected with empty vector LV-003 and BR-V108, as negative control; shCDK1 + ZNF655 indicates simultaneously downregulated CDK1 and upregulated ZNF655 in PANC-1 cells. The presented results were representative of experiments repeated at least three times. Data was represented as mean ± SD. *P < 0.05, **P < 0.01, ***P < 0.001.

Functional recovery experiments were conducted to clarify whether there was a synergistic effect between ZNF655 and CDK1 in pancreatic cancer. PANC-1 cells overexpressing ZNF655 lead to a promotion in proliferation and migration, as well as an inhibition in apoptosis (Fig. 4A–D). Furthermore, the overexpression of ZNF655 and the knockdown of CDK1 (shCDK1 + ZNF655) were established in PANC-1 cells (Fig. S2F). The malignant progression of PANC-1 cells in shCDK1 + ZNF655 group was significantly inhibited compared with ZNF655, which was characterized by weaken proliferation (*P* < 0.001), impeded migration and enhanced apoptosis (*P* < 0.001) (Fig. 4A–D). Taken together, the loss/gain-of-function assays demonstrated that knockdown of CDK1 could alleviate the promoting effects of ZNF655 overexpression in PANC-1 cells.

### Knockdown of ZNF655 suppresses tumor growth in the mouse xenograft model

**Fig. 5:**
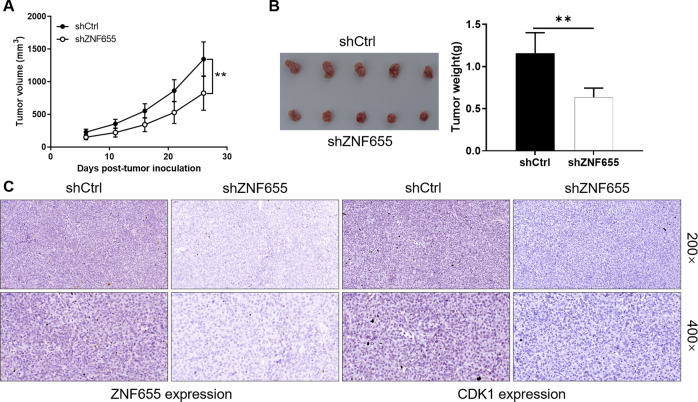
Knockdown of ZNF655 attenuates tumor formation of pancreatic cancer in vivo. **A** Post injection of SW1990 cells for 7 days, the tumor volume in mice was measured. **B** Mice were sacrificed at day 26 post injection, and the tumor weight was measured and photographed. **C** The expression of ZNF655 and CDK1 in mice tumor tissues was detected by IHC staining. Data was represented as mean ± SD. *P < 0.05, **P < 0.01, ***P < 0.001.

To further verify the effects of ZNF655 in vivo, SW1990 cells were injected subcutaneously into the BALB/c nude mice and divided into shCtrl group and shZNF655 group. Monitoring of mice 26 days after the cell injection showed that that the tumor volume of shZNF655 group was significantly weaker than that of the control group (*P* < 0.01) (Fig. 5A). Consistently, the tumor weight of shZNF655 group was significantly lighter than that of the control group (Fig. 5B). Moreover, IHC staining performed on tumor sections from xenografts showed that ZNF655 and CDK1 expression level was markedly reduced in the ZNF655 knockdown group compared with the control group (Fig. 5C). Collectively, these results highlighted that reduced expression of ZNF655 suppressed the tumor growth in vivo.

## Discussion

A significant finding of this study is the identification of a promoting role of ZNF655 in human pancreatic cancer. Previous evidence has shown that the development and progression of human pancreatic cancer is a complex, multifactorial process with multiple genetic abnormalities [3]. The characteristic of early pancreatic intraepithelial tumors is that mutations in the oncogene KRAS lead to the activation of the RAS/RAF and PI3K/AKT signaling pathways, which are intracellular signaling pathways that regulate the cell cycle [32]. In this study, we found that that malignant progression of ZNF655 knockdown pancreatic cancer cells was significantly inhibited, which was characterized by weaken proliferation, impeded migration, arrested cell cycle in G2 and enhanced apoptosis. In fact, ZNF655 knockdown by shRNA specifically enhanced the apoptotic sensitivity of pancreatic cancer cells, while ZNF655 overexpression resulted in reduced apoptotic capacity. In this way, why a single TF perturbs so many different tumor-promoting features still stands. we found that ZNF655, a TF, can affect a variety of functions in a variety of cancers. For example, Chen et al., indicated that ZNF655 promotes the progression of glioma through transcriptional regulation of AURKA [33]. Teng et al., demonstrated that the decrease of ZNF655 expression led to the inhibition of the malignant behaviors of non-small cell lung cancer, which was manifested by weakened proliferation, increased sensitivity to apoptosis, cycle repression at G2 and weakened migration [34]. Yang et al., identified role of ANKHD1/LINC00346/ZNF655 feedback loop in regulating the glioma angiogenesis via staufen1-mediated mRNA decay [35]. In general, ZNF655 may affect multiple functions of cancer by targeting certain genes.

Furthermore, ZNF655 knockdown resulted in downregulation of CCND1 expression and inhibition of PI3K/AKT signaling pathway in pancreatic cancer cells. Additionally, the characteristics of early lesions also include the inactivation of the tumor suppressor gene CDKN2A and the overexpression of the oncogene CDKN1A, which promote the transition of the cell cycle from the G1 phase to the S phase [36]. In later lesions, two key tumor suppressor genes, Smad4 and TP53, were inactivated. The inactivation of Smad4 and its coding protein blocks the typical downstream signaling of transforming growth factor β (TGFβ) receptor, while the inactivation of TP53 and its coding protein P53 promote the progression of cell cycle, survival and inhibition of apoptosis [37, 38]. Furthermore, ZNF655 knockdown upregulated the expression of Bax, BIM, Caspase3, cytoC, IGFBP-6, p21, p27 and p53, while downregulated the expression of Survivin, IGFBP-2 and STNF-R2 in PANC-1 cells. ZNF655 knockdown can promote the apoptosis of pancreatic cancer cells, which required the co-participation of a series of pro-apoptotic and anti-apoptotic factors. Indeed, ZNF655 may affect tumor progression through multiple pathways. Given that apoptosis is one of the biological phenotypes of cancer, this study focused on the effect of ZNF655 knockdown on apoptosis and the expression of apoptosis-related proteins. Of course, it is impossible to study all biological pathways related to cancer in one project. Therefore, we will delve into other biological processes related to cancer in future work.

The downstream mechanism of ZNF655 regulating pancreatic cancer progression was preliminarily investigated and found that knockdown of ZNF655 downregulated CDK1. To our knowledge, CDK1 is responsible for driving cell division and coordinating the transition from G2 phase to mitosis [39]. Uncontrolled proliferation signaling leads to dysregulation of the cell cycle, which is considered a hallmark of cancer [40]. Recently, it has been reported that that CDK1 is a typical cell cycle regulator and has promoted the progression of pancreatic cancer [30]. On the other hand, NCAPD2 could affect tumor cell proliferation, apoptosis, migration and invasion by targeting CDK1 in breast cancer [41]. Consistently, we stably knocked down CDK1 in PANC-1 cells using lentiviral shCDK1 and the loss-of-function results showed a significant inhibition in the proliferation and migration of CDK1-knocked-down PANC-1 cells. Additionally, knockdown of CDK1 alleviated the promoting effects of ZNF655 overexpression in pancreatic cancer cells. As a consequence, ZNF655 promoted the malignant behavior of pancreatic cancer cells through CDK1.

Combined with our data, we inferred that there may be a connection between ZNF655 and CDK1. Moreover, it was reported that E2F1 binds to the upstream region of CDK1 to accelerate transcription and upregulated its protein expression [31]. Interestingly, the present study indicated that there was interaction between ZNF655 and E2F1. In addition, dual-luciferase reporter experiment and Ch-IP assay further confirmed that overexpression of ZNF655 induced E2F1 to be recruited to the CDK1 promoter region. Mechanically, these data suggested that ZNF655 facilitated malignant behaviors of pancreatic cancer cells via promoting the binding of E2F1 to CDK1 promoter.

ZNF655 expression was significantly elevated in human pancreatic cancer and possessed clinical value in predicting poor prognosis. ZNF655 facilitated malignant behaviors of pancreatic cancer cells via promoting the binding of E2F1 to CDK1 promoter, which may contribute to the development of promising targets for tumor diagnosis and treatment.

## Supplementary information

Supplementary materials

## Data Availability

The data used and analyzed during the current study are available from the corresponding author on reasonable request.
