# Supplementary material for: ZNF655 accelerates progression of pancreatic cancer by promoting the binding of E2F1 and CDK1
Source: Oncogenesis. 2022 Aug 4;11(1):44. doi: 10.1038/s41389-022-00418-2 (PMC9352668; doi:10.1038/s41389-022-00418-2)
Supplement: Supplementary file 1 — Supplementary materials [file 41389_2022_418_MOESM1_ESM.docx]

| Gene | Upstream primer sequence | Downstream primer sequence | Amplified fragment size (bp) |
| --- | --- | --- | --- |
| GAPDH | AAATACCAGCCCAGGAAGCA | AAACTGAGGCTCTGGGGACA | 121 |
| ZNF655 | AAATACCAGCCCAGGAAGCA | AAACTGAGGCTCTGGGGACA | 92 |
| CCDC88A | TTCCCAAAGTACCTCACT | TTTAGGTCTTCTCGCTCT | 105 |
| HMGB1 | CAGATGCTTCAGTCAACTTCTCAG | CCGCTTTTGCCATATCTTCA | 105 |
| CCNB2 | AAGTTCCAGTTCAACCCACCA | GCAGAGCAAGGCATCAGAAAA | 176 |
| NUF2 | GGGTGAATGACTTTGAGACTGCT | GCTGCTTGAACTCTTCTTGCTCTT | 249 |
| CDK1 | CCATACCCATTGACTAACTAT | ACCCCTTCCTCTTCACTTTC | 150 |
| CDC42EP3 | AGCAGTCTGTTGGAGAATGGG | AGGAGGGAACCTGTAAGGTCAG | 227 |
| HMGB3 | ACCGTCTGGATTCTTCCTGTT | GATGTAAGGCTGCTTTTCACTG | 142 |
| MDM2 | GGTGAGGAGCAGGCAAATGT | CGAAGCTGGAATCTGTGAGGT | 82 |
| SKP2 | ATAGAAGTGTCCACCCTCCACG | CACCCAGAAAGGTTAAGTCGC | 143 |
| KIF18A | AGAAATCCTGAACTGCTTGT | ATGGCACTGTTGTTGGTAGA | 115 |
| NEK2 | GAAGGAATGCCACAGACGAAGT | CAAGCAGCCCAATGACCAGATA | 244 |
| CASC4 | CCAAGGACAGAGTCAGTGATTTC | CTACGTTACCATCATCCCCATT | 126 |
| PLAC8 | CACATTTTGTTTCCCGTGCCT | TCATTGCGACGCTTGTTCC | 83 |
| KIF2C | AGATGGAAGCCTGCTCTAACGG | GCTGGACATCTGGGAAGACAGT | 83 |
| GDF15 | GCAAGAACTCAGGACGGTGA | TGGAGTCTTCGGAGTGCAAC | 146 |
| ANTXR1 | AGGTGCTAAGTTGGAAAAGGC | TGTTGAGATTTCGCGGCTC | 86 |
| RDM1 | GCCCATCCTGGTTTCTATGC | GGCTTGATGTTGAACTGCCTTA | 150 |
| KDM1B | CTTTCTACCAGCCCAATG | TGCGAGGATGAGGTTTCT | 137 |
| NRAS | AAACCTCAGCCAAGACCAGA | AACCCTGAGTCCCATCATCAC | 114 |

Table S1. Primer sequence for PCR

Table S2. Antibodies used in western blotting and Co-IP

| Primary antibodies | Dilution in WB | Source species | Company | Catalog No. |
| --- | --- | --- | --- | --- |
| ZNF655 | 1:1000 | Rabbit | Invitrogen | PA5-56183 |
| CDK1 | 1:1500 | Rabbit | Abcam | ab133327 |
| HMGB3 | 1:1000 | Rabbit | Abcam | ab75782 |
| MDM2 | 1:1000 | Rabbit | Abcam | ab178938 |
| PLAC8 | 1:1000 | Rabbit | CST | 13885 |
| AKT | 1:1000 | Rabbit | CST | 4685 |
| p-AKT | 1:500 | Rabbit | R&D | AF887-sp |
| CCND1 | 1:1000 | Rabbit | CST | 2978 |
| PIK3CA | 1:1000 | Rabbit | Abcam | ab40776 |
| GAPDH | 1:3000 | Rabbit | Bioworld | AP0063 |
| Primary antibodies | Dilution in Co-IP | Source species | Company | Catalog No. |
| DYKDDDDK Tag | 1:50/1:1000 | Rabbit | CST | 14793 |
| ZNF655 | 1:2000 | Rabbit | Novus | NBP1-78732 |
| CDK1 | 1:3000 | Rabbit | Abcam | ab133327 |
| GAPDH | 1:30000 | Mouse | Proteintech | 60004-1-lg |
| Secondary antibody | Dilution |  | Company | Catalog No. |
| HRP Goat Anti-Rabbit IgG (WB) | 1:3000 |  | Beyotime | A0208 |
| HRP Goat Anti-Rabbit IgG (Co-IP) | 1:3000 |  | Beyotime | A0216 |


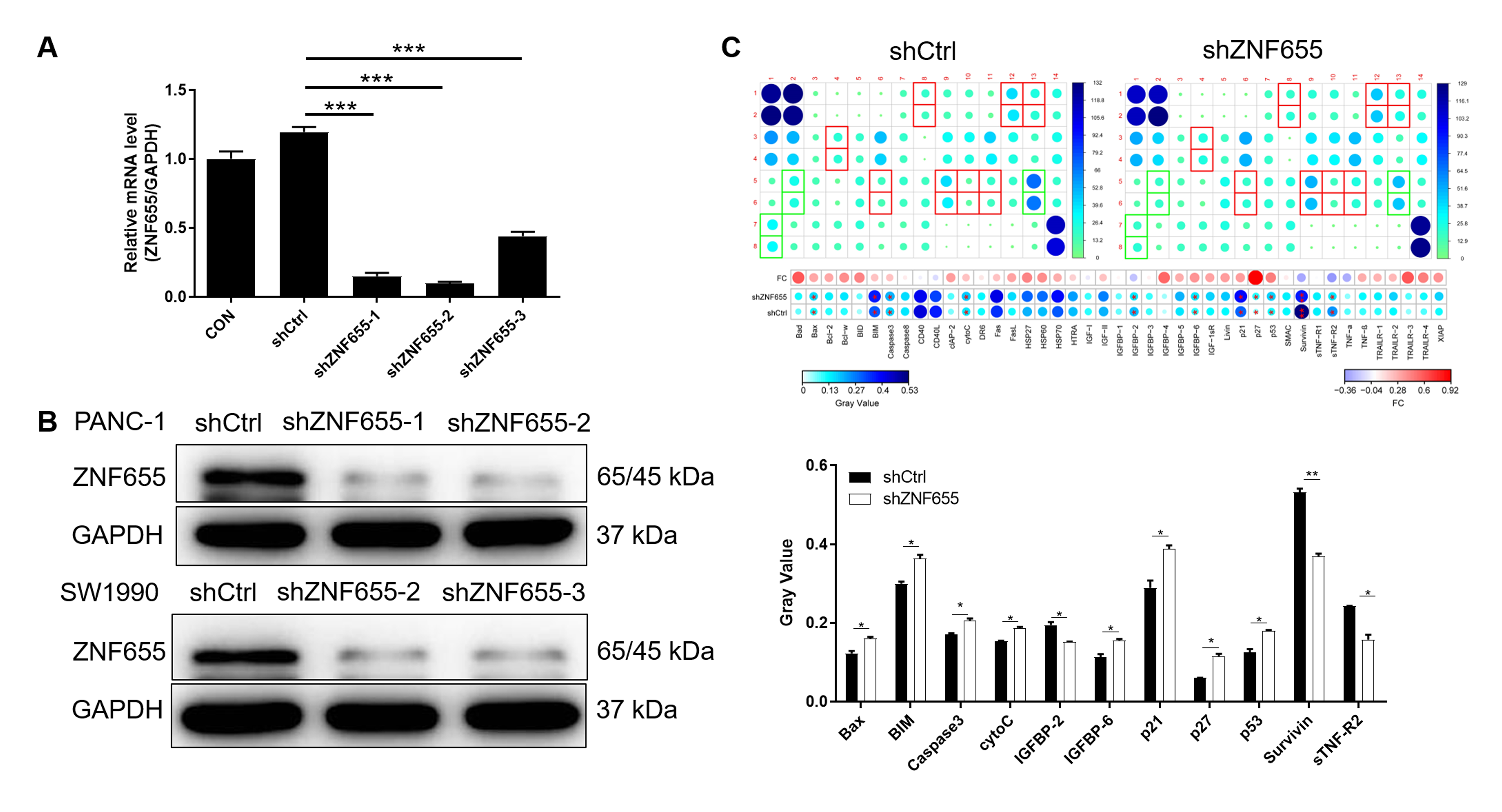


**Fig. S1. The establishment of ZNF655 knockdown pancreatic cancer cells and the detection of apoptosis-related protein expression.** (A) The sequences of shRNA targeting ZNF655 (shZNF655-1/2/3) were synthesized detected the ZNF655 expression. (B) The protein expression of ZNF655 in PANC-1 and SW1990 cells was measured after the lentivirus shZNF655-1 and shZNF655-2 were transfected. (C) The expression of 43 apoptosis-related regulators of PANC-1 cells after knockdown of ZNF655 was detected using apoptotic antibody array membrane. The presented results were representative of experiments repeated at least three times. Data was represented as mean ± SD. *P < 0.05, **P < 0.01, ***P < 0.001.


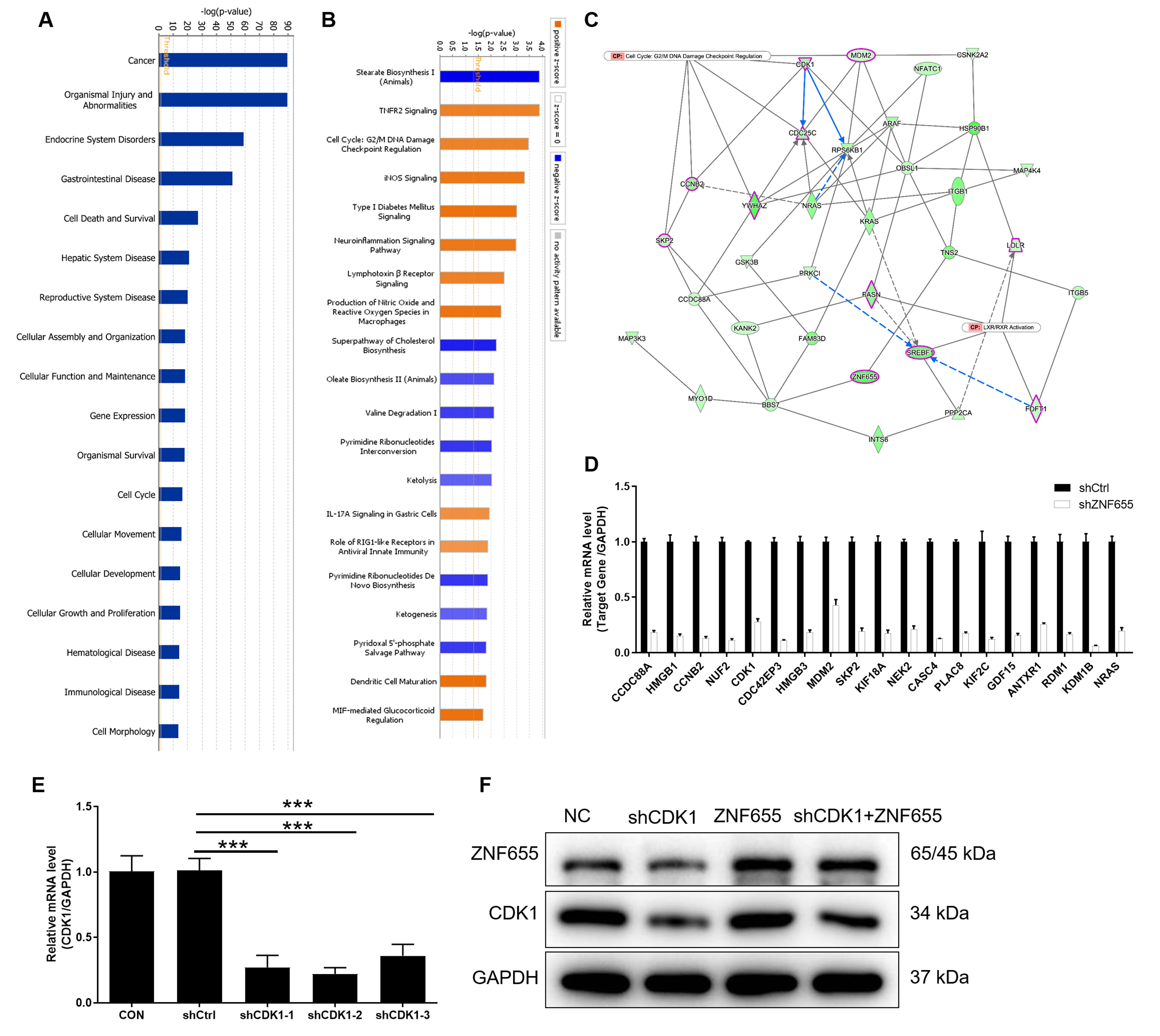


**Fig. S2. Screening of downstream targets of ZNF655 in the regulation of pancreatic cancer.** (A) The enrichment of the DEGs in IPA disease and function was analyzed by IPA. (B) The enrichment of the DEGs in canonical signaling pathways was analyzed by IPA. (C) Interaction network diagram among DEGs was analyzed by IPA. (D) The expression of several selected DEGs of PANC-1 cells after knockdown of ZNF655 was measured by qPCR. (E) The sequences of shRNA targeting CDK1 (shCDK1-1/2/3) were synthesized detected the CDK1 expression. (F) The protein expressions of ZNF655 and CDK1 were detected in PANC-1 cells transfected with lentivirus (shCDK1, ZNF655, shCDK1+ZNF655), respectively.
